# Draft genome sequence of *Trichococcus flocculiformis* DSM 2094^T^ isolated from bulking sludge in Germany

**DOI:** 10.1128/mra.01293-24

**Published:** 2025-03-25

**Authors:** Asema Amin, Jessica Mojica, Adrian Sanchez, Ruediger Pukall, Markus Göker, Kurt LaButti, Tanja Woyke, Rekha Seshadri, Tricia A. Van Laar

**Affiliations:** 1 Department of Biological Sciences, California State University, Stanislaus, Turlock, California, USA; 2 Leibniz Institute DSMZ--German Collection of Microorganisms and Cell Cultures GmbH, Braunschweig, Germany; 3 DOE Joint Genome Institute, Lawrence Berkeley National Laboratory 1666, Berkeley, California, USA; SUNY College of Environmental Science and Forestry, Syracuse, New York, USA

**Keywords:** wastewater treatment

## Abstract

Here we describe the draft genome sequence of *Trichococcus flocculiformis* DSM 2094^T^ (strain Echt) isolated from bulking sludge in Germany. The genome is 2,978,824 bp and contains genes encoding biofilm and capsule formation, which might contribute to the bulking phenomenon associated with this isolate.

## ANNOUNCEMENT

Sludge bulking, caused by the overgrowth of filamentous bacteria, disrupts solid settling and reduces effluent quality in wastewater treatment plants (1). A gram-positive, filamentous bacterium was isolated from a plant experiencing sludge bulking in Echterdingen, Germany, by plating sludge on UA medium followed by incubation at 25°C (2). The organism was named *Trichococcus flocculiformis* DSM 2094^T^. Other isolates from sludge closely match *T. flocculiformis* DSM 2094^T^ morphologically and biochemically (3). A comparative 16S rRNA gene sequence analysis identified many isolates as either different strains of *T. flocculiformis* or different species within the *Trichococcus* genus (3).

The genome of *T. flocculiformis* DSM 2094^T^ was sequenced as part of the Genomic Encyclopedia of Bacteria and Archaea (GEBA) effort to sequence type strains and newly discovered organisms, particularly those representing increased phylogenetic diversity (4, 5). The bacterium was grown in DSMZ medium 183 (https://mediadive.dsmz.de) at 25°C by Leibniz Institute DSMZ. Genomic DNA was isolated using the MasterPure Gram-positive DNA extraction kit (Epicentre). DNA was sheared to 300 bp using the Covaris LE220, and the size was selected using AMPure XP SPRI beads (Beckman Coulter). The fragments were treated with end-repair, A-tailing, and ligation of Illumina compatible adapters (IDT, Inc) using the KAPA-Illumina library creation kit (KAPA biosystems). The library was quantified using the KAPA Biosystems next-generation sequencing library qPCR kit (Roche). The quantified library was prepared for sequencing utilizing a TruSeq paired-end cluster kit, v3, and Illumina’s cBot instrument. Sequencing was performed on the Illumina HiSeq 2500 sequencer using HiSeq TruSeq SBS sequencing kits, v3, following a 2 × 150 indexed run recipe.

A total of 10,454,276 reads and 1,510 Mb were sequenced, resulting in 460× coverage. Reads were filtered using the JGI-developed program, DUK (6). The genome was assembled using Velvet v 1.2.07 (7), wgsim v 0.3.0 (8), and Allpaths-LG v r46652 (9). Specific parameters were velveth: 63 –shortPaired; velvetg: –very clean yes –exportFiltered yes –min contig lgth 500 –scaffolding no –cov cutoff 10; wgsim: –e 0–1 100–2 100 –r 0 –R 0 –X 0, and Allpaths-LG: PrepareAllpathsInputs: RunAllpathsLG: CheckM2 v 1.0.2 (10) estimated that the genome was 99.96% complete with 0.83% contamination. The genome was annotated with the standard DOE-JGI Microbial Genome Annotation Pipeline (MGAP v.4) (11) as part of IMG/M (12).

The draft genome of *T. flocculiformis* DSM 2094^T^ is 2,978,824 bp with 45.58% G + C content. The assembly consists of 110 scaffolds with an N50 value of 47,460 bp (Table 1). The genome encodes 2,892 protein-coding genes, 9 rRNA genes, and 47 tRNA genes. The Virulence Factor Database (VFDB) predicted the presence of genes involved in biofilm formation, capsule production, and adhesion, all of which could contribute to bulking sludge (13). As this organism was isolated from sewage, we used ResFinder v 4.5.0 (14) to mine the genome for any antimicrobial resistance cassettes, but none were identified. CRISPRCas Finder v 1.1.2 (15) found one potential CRISPR region in the bacterial genome. Further exploration of the *T. flocculiformis* DSM 2094^T^ genome and those of other sludge-bulking bacteria holds great potential for improving wastewater treatment processes worldwide.

**TABLE 1:** Genome features of *T. flocculiformis* DSM 2094^T^

Feature	Finding
Total scaffold sequence length (bp)	2,978,824
No. of scaffolds	110
Scaffold N*_50_* (kbp)	47,460
Genome coverage	776×
GC content (%)	45.58
No. of genes	2,969
No. of protein-coding genes	2,892
No. of rRNA	9
No. of tRNA	47

## Data Availability

The genome sequence was GenBank under the accession number FOQC00000000. The raw reads have been deposited in the NCBI SRA under the accession number SRX2134213. Additional data can be explored or downloaded from the JGI Integrated Microbial Genomes with Microbiomes (IMG/M) portal using the taxon ID 2619618836.
